# How time gets spatial: factors determining the stability and instability of the mental time line

**DOI:** 10.3758/s13414-023-02746-w

**Published:** 2023-07-19

**Authors:** Gabriele Scozia, Mario Pinto, Michele Pellegrino, Silvana Lozito, Lorenzo Pia, Stefano Lasaponara, Fabrizio Doricchi

**Affiliations:** 1 Dipartimento di Psicologia, Università degli Studi di Roma ‘La Sapienza’, Roma, Italy; 2 PhD program in Behavioral Neuroscience, Università degli Studi di Roma ‘La Sapienza’, Roma, Italy; 3 Department of Psychology, University of Turin, Turin, Italy; 4 Fondazione Santa Lucia IRCCS, Roma, Italy

**Keywords:** Temporal processing, Spatial cognition, Embodied perception

## Abstract

Left-to-right readers classify faster past events with motor responses on the left side of space and future events with responses on the right side. This suggests a left-to-right spatial organization in the mental representation of time. Here, we show that the significance and reliability of this representation are linked to the joint use of temporal and spatial codes in the task at hand. In a first unimanual Go/No-Go Implicit Association Test (IAT), attending selectively to “past” or to “future” words did not activate corresponding “left” or “right” spatial concepts and vice versa. In a second IAT, attending to both temporal (i.e., “past” and “future”) words and spatial targets (i.e., “left” and “right”) pointing arrows produced faster responses for congruent rather than incongruent combinations of temporal and spatial concepts in task instructions (e.g., congruent = “Go with past words and left-pointing arrows”; incongruent = “Go with past words and right-pointing arrows”). This effect increased markedly in a STEARC task where spatial codes defined the selection between “left-side” and “right-side” button presses that were associated with “past” and “future” words. Two control experiments showed only partial or unreliable space–time congruency effects when (a) participants attended to superordinate semantic codes that included both spatial “left”/”right” or temporal “past/future” subordinate codes; (b) a primary speeded response was assigned to one dimension (e.g., “past vs. future”) and a nonspeeded one to the other dimension (e.g., “left” vs. “right”). These results help to define the conditions that trigger a stable and reliable spatial representation of time-related concepts.

**Supplementary information:**

The online version contains supplementary material available at 10.3758/s13414-023-02746-w.

People use space to think and talk about the flow of time (Bonato et al., 2012; Boroditsky, 2001; Tversky, 1991; for review, see Núñez & Cooperrider, 2013; Oliveri et al., 2009). A salient example of this conceptual association is provided by the STEARC effect (Spatial–Temporal Association of Response Codes; Ishihara et al., 2008). The STEARC shows that participants belonging to left-to-right reading cultures are faster at classifying short-time durations with motor responses in the left side of space and long durations with responses on the right side (Conson et al., 2008; Ishihara et al., 2008; Scozia et al., 2023; Vallesi et al., 2008). The STEARC effect is also observed when high-level semantic processing is required to classify words or sentences as referring to the past or the future (e.g., “yesterday” vs. “tomorrow”; Santiago et al., 2007; Torralbo et al., 2006). This latter finding extends to pictorial stimuli so that following the presentation of a sequence of pictures or a video depicting a short story, observers are faster at classifying pictures and video frames from the beginning of the story with left-side button presses and pictures or frames from the end of the story with right-side presses (Santiago et al., 2010).

The STEARC effect supports the idea that humans use a mental spatial device, the “mental time line” (MTL), to represent the flow of time (Bonato et al., 2012). Different types of MTLs have been described, grounded on different types of sensorimotor and cultural experiences (Casasanto & Boroditsky, 2008; Núñez & Sweetser, 2006; Pitt & Casasanto, 2020). Horizontal MTLs would be oriented according to acquired cultural scanning and reading habits that determine specific correlations between points in space and time (Pitt & Casasanto, 2016, 2020). For example, inspecting from left-to-right means leaving left-side items more in the past the more an item is placed at the beginning of the inspection. For this reason, a left-to-right mapping of time is found in Western individuals, while an opposite right-to-left mapping is found in Arabic or Israelian ones (Fuhrman & Boroditsky, 2010; Ouellet et al., 2010).

Sagittal MTLs with the past placed in backward and the future in forward space are grounded on biologically constrained sensorimotor experiences linked to forward locomotion that make us “leave the past behind” (Sell & Kaschak, 2011; Teghil et al., 2021; Ulrich et al., 2012; though see an interesting exception in the Aymara population; Núñez & Sweetser, 2006). Finally, the use of vertical time lines, with less recent events on the top and future events on the bottom side of the line, seems linked both to the writing-style direction of specific cultures and to how frequently a vertical representation of time is adopted in graphical tools in different cultures (e.g., calendars in Western left-to-right reading cultures; Starr & Srinivasan, 2021)

In a recent meta-analysis of the literature, von Sobbe et al. (2019) pointed out that the task relevance of the temporal dimension has a crucial impact in triggering the spatial representation of time. These authors identified three main different types of temporal tasks. First, there are tasks in which “time is task-relevant” as participants have to explicitly categorize the temporal reference of the stimulus. Second, there are tasks in which “time is task-irrelevant” as participants have to categorize a feature of the stimulus conveyed through time-related material but not temporal “per se.” For example, participants have to decide whether a past-related or a future-related sentence is sensible so that, in this case, the influence of the implicit processing of temporal information on task performance is tested (Ulrich & Maienborn, 2010). Finally, there are “temporal priming” tasks, in which temporal cues eventually favour or disfavour the processing of spatial targets or the selection of spatially defined responses. For example, in participants who have to classify with left versus right motor response whether a visual target has appeared in the left or right side of space, past-related word cues preceding the target facilitate detections in the left side of space and future-related cues in the right side (Weger & Pratt, 2008, Experiment 2a).

Most important for the aims of the present study, in the same review von Sobbe et al., (2019) pointed out that in the large majority of studies, space was incorporated into the task by contrasting “left vs. right,” “up vs. down”, or “back vs. forward” spatial positions for response choice. Motor responses consisted of button presses or directional hand–finger movements (e.g., moving a joystick). In a minority of cases, spatial codes were introduced in the task by manipulating the position of the temporal material. For example, Torralbo et al. (2006) presented time words related to the past or the future to the left o to the right of a head silhouette. Walker et al. (2014) presented auditory private temporal events that had to be verbally classified as belonging to the past or the future (deictic condition) or as happening earlier or later than another private event (sequential condition). Auditory temporal events were presented by one of four speakers positioned to the left, right, and in front or in back of participants.

The present study aims to expand on the insights offered by the review by von Sobbe et al. (2019) and investigate more in depth how primal the link between the representation of space and the representation of time is in the cognitive system and the brain. In particular, we would like to provide empirical answers to questions like, Does the selective activation of the concept “left” implies the automatic and simultaneous activation of concepts like “past” or “short duration”? Is the simultaneous presence of contrasting “left vs. right” spatial response codes in the task necessary to trigger the spatial mental organization of “past vs. future” temporal features associated with response choices? More in general, which are the cognitive and behavioural conditions that determine the co-activation and association between time-related and space-related concepts?

Insights into these problems are offered by recent investigations (Pinto et al., 2019a, b, 2021a, b) in the functional bases of the spatial representation of number magnitudes, the space–number association (SNA; Cipora et al., 2020; Fattorini et al., 2016; Fischer & Shaki 2014; Shaki & Fischer, 2018). The prototypical empirical example of the association between space and number representations is offered by the SNARC effect (spatial number association of response codes; Dehaene et al., 1993). In left-to-right readers, the SNARC consists of a faster classification of small numbers with motor responses in the left side of space and large numbers with responses on the right side. This effect is conventionally interpreted as deriving from the congruency between the left/right position of motor responses and the inherent and task-independent left-to-right positioning of numbers along a horizontal mental number line (MNL) that is organized according to cultural reading and scanning habits (note that the direction of the MNL and the SNARC is reversed in right-to-left reading cultures; Pitt & Casasanto, 2020; Shaki et al., 2009).

Nonetheless, the results of some recent investigations offer a reinterpretation of the SNARC. These investigations were run with the Implicit Association Test (IAT; Nosek & Banaji, 2001). In the IAT, Arabic numbers ranging from 1 to 9, and horizontal left- or right-pointing arrows are alternated at central fixation. In a first experimental condition, defined as “single code” (SC), participants must provide go/no-go unimanual motor responses based only on the magnitude of numerical targets (e.g., Go when a number is lower than 5 and whenever an arrow is presented) or based only on the direction of the arrow (e.g., Go when an arrow points left and whenever a number appears). Using the IAT, Pinto et al. (2019a, b, 2021a, b) have demonstrated that responding only to numbers smaller than 5 while responding to all arrows, independently of their direction, does not speed up responses to left-pointing arrows compared with right-pointing ones. This result is maintained when the processing of arrows is forced by intermixing additional trials with nontarget distracters (i.e., a visual dot) that requires no motor response. The same happens when participants attend only to a specific arrow direction while responding to all numbers independently of their magnitude. In contrast, a reliable SNA is found when instructions require discriminating, in different trials of the same task, both the magnitude of numerical targets and the direction of arrow targets (Pinto et al., 2019a, b, 2021a, b). In this “joint code” (JC) condition, faster RTs are observed for instructions that combine in a congruent way the number magnitude and its position on the MNL (e.g., “Go when a number is smaller than 5 and when an arrow points to the left”) as compared with incongruent instructions (e.g., “Go when a number is smaller than 5 and when an arrow points to the right”). These findings show that reliable and stable horizontal MNLs are generated only when left/right spatial codes are jointly activated together with small/large number magnitude codes. Taken together, these findings suggest that rather than arising from the congruency between the left/right position of motor responses and the inherent and task-independent position of numbers along the MNL, in the SNARC task, it is the very use of contrasting left–right spatial codes for response selection that triggers the generation of a corresponding spatially organized MNLs with small numbers positioned on the left side and large ones on the right side. Consequently, when no contrasting left-right spatial codes are used, no reliable and stable MNL is generated (Pinto et al., 2019a, b, 2021a, b).^1^ We note that this conclusion on the SNA is in line with the more general idea that von Sobbe et al. (2019) have advanced for the space–time association (STA), that “activating a mental timeline will only happen in those situations in which there is a gain to cope with that particular situation and its requirements.”

Based on the above-summarized findings in the domain of the SNA, here we wished to investigate the functional basis of the STA more in depth. To this aim, we used an IAT (Nosek & Banaji, 2001) with intermixed left/right-pointing arrow targets and linguistic temporal targets. Linguistic targets consisted of a list of 20 “past” (e.g., “yesterday”) and “future” (e.g., “tomorrow”) words (verbs and adverbs) derived from the study by Santiago et al. (2007). In a first series of three experiments, the same healthy adult participants performed a unimanual IAT go/no-go task in the single code and joint code conditions and a conventional bimanual STEARC task. Then, in two additional control experiments, we investigated (a) to which degree temporal “past vs. future” and spatial “left vs. right” conceptual dichotomies must be explicitly activated by task instructions to generate mental time lines when both temporal and spatial codes are relevant to the performance of the task; (b) to which degree temporal and spatial concepts must compete in the selection of speeded motor go responses to generate a significant and reliable MTL.

The fundamental prediction of our study is that if the STA derives from an inherent association between spatial concepts (e.g., “left”) and corresponding temporal concepts (e.g., “past”), then in an IAT, the mere and selective activation of one spatial concept (e.g., “left”) should consistently and reliably facilitate the activation and classification of the associated “past” temporal concept. Alternatively, if the positioning of “past” and “future” temporal concepts on the left and right side of an MTL depends on the simultaneous presence of contrasting “left vs. right” response codes in the task at hand, then a significant and reliable STA should be found only in (a) IATs that require discriminating, in different trials of the same task, both the “left vs. right” direction of arrow targets and classifying “past vs. future” word targets; (b) conventional STEARC tasks where contrasting “left vs. right” spatial response codes are associated to contrasting “past vs. future” temporal stimulus codes.

## General methods (Experiments 1–3)

### Participants

To determine the number of participants, we ran an a priori power analysis (G*Power; Faul et al., 2007) using the effect size *f*(U) = 0.4388 derived from the previous study of Santiago et al. (2007). This analysis showed that 27 participants would be needed to have a power of .90, considering an alpha of .05 (two-sided) of statistical significance for repeated-measures within-factors analyses of variance (ANOVAs).

Based on this preliminary analysis, we tested 28 healthy adult participants. Each participant performed three different experiments included in the study (20 F and 8 M, mean age = 23.57 years, *SD* = 3.39; all participants were right-handed Italian native speakers). To avoid “carryover” effects that can take place in within-participants designs, the order of tasks administration followed that of the increasing complexity of task instructions. As an example, in the first experiment (see below), in different blocks of trials, unimanual responses were regulated by the consideration of a “single” time or space code (e.g., “Go when an arrow points left and Go to all words”). In the second experiment, unimanual responses were regulated by the “joint” association of a space code with a time code (e.g., “Go when an arrow points left and when a word indicates a past event”). Finally, in the third experiment, the “joint” association of a space with a time code determined the selection of a left vs. right side motor response (e.g., “Push the left button when a word indicates the past and the right button when it indicates the future”). It is clear, for example, that performing the second “joint” experiment first would activate the explicit association between a space and a time code and that this association can be “carried over” to the performance of the first “single” code experiment, that is aimed at testing whether the activation of a single space or time codes produces the implicit activation of a corresponding and congruent time or space code, respectively. Vice versa, performing first the single code experiment does not activate the conceptual space–time association and does not produce corresponding carryover effects on the performance of the second “joint” code experiment. Following the same line of reasoning, we point out that the STEARC effect that in the present study was tested in the third final experiment is a very consolidated and replicated finding and that, therefore, the replication of this effect can hardly be attributed to the previous performance of the second unimanual experiment with the joint use of space and time codes.

As a further control for “carryover” effects, we ran a series of correlation and regression analyses among the individual performance (i.e., space–time congruency effects) in the first three experiments.

### Apparatus

Due to COVID pandemic restrictions, experiments were administered through the open-source software OpenSesame (https://osdoc.cogsci.nl/3.3/; Mathôt al., 2012), imported on a Jatos Server (https://www.jatos.org/). Participants accessed the experiment using a General Multiple Worker link. Participants were instructed to run the experiment in a quiet and isolated room and wear in-ear plug headphones to reduce environmental noise sources. They were also asked to keep their head positioned at a viewing distance of 60 cm from the screen. All participants had a normal or corrected-to-normal vision and were naïve to the aim of the study. Instructions for all experiments were provided during individual audio/video calls with one of the experimenters. A training block that included 24 trials was administered before the experimental session and always corresponded to a shortened version of the first experimental block.

### Stimuli

Here, we describe the stimuli used in each of the three experiments included in the study. Instructions for the three experiments are detailed in the corresponding sections.

**Fig. 1:**
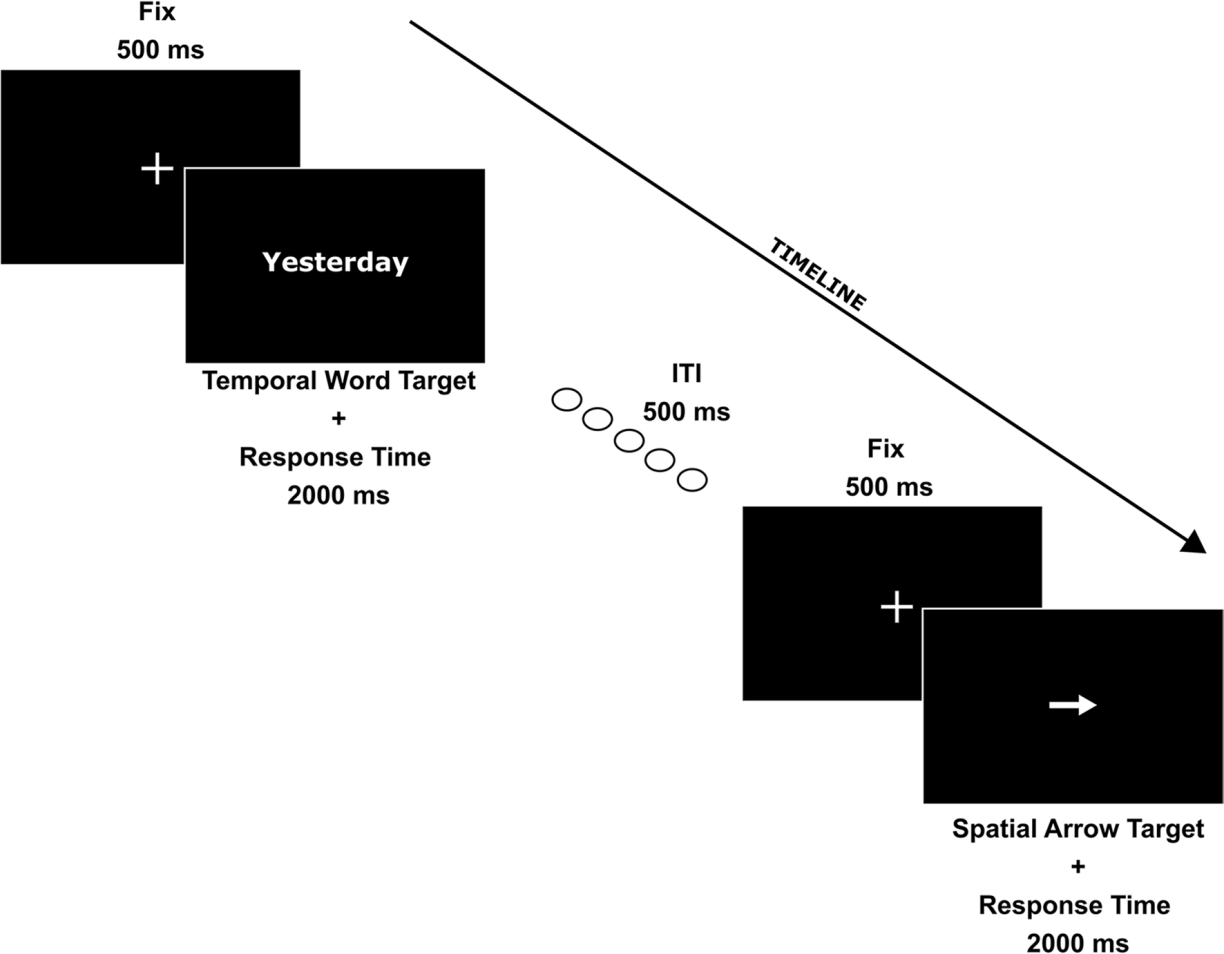
Examples of two consecutive trials in the go/no-go task of Experiments 1 and 2. The first is a temporal trial with a word-target (the past verb “Yesterday”). The second is a spatial trial with an arrow-target (arrow pointing to the right in this example)

A go/no-go task was administered in Experiments 1 and 2. Each go/no-go trial started with the 500-ms presentation of a central fixation cross (1.5° × 1.5°). At the end of this delay, go arrow-target (size = 2° × 0.8°) pointing to the left or right, or go linguistic temporal target (verbs and adverbs explicitly referred to the past or future; see lists in Supplementary Material) replaced the central fixation cross. In Experiment 1, temporal go words were also intermixed with no-go nonwords, and arrow-targets were intermixed with no-go white circles (diameter = 2°; see below). Target stimuli remained available for response for 2,000 ms. Participants provided manual go responses by pressing the central spacebar on the computer keyboard. The fixation cross and the targets were white on a black background. The intertrial interval was 500 ms (see Fig. 1). In Experiment 1 and Experiment 2, four blocks of trials with different go/no-go instructions were administered during a single experimental session. The order of blocks was counterbalanced among participants. A short break was allowed between blocks.

In Experiment 3, a conventional bimanual STEARC task was administered. At variance with Experiments 1 and 2, only temporal word targets were presented in this task (i.e., words referring to the past or the future). Participants were asked to respond by using one out of two response buttons on the keyboard: one on the left (x) and one on the right side of the keyboard (m). The trial events’ timing and sequence were similar in Experiment 1 and Experiment 2. The order of blocks was counterbalanced among participants.

## Experiment 1: Single code (SC)

In the first go/no-go experiment, hereby defined “single code” task (SC), participants responded to targets according to instructions that required the discrimination of the left/right direction of arrows without requiring the discrimination of the past/future temporal targets or vice versa, the discrimination of the past/future temporal targets without requiring the discrimination of the left/right direction of arrows. The aim of the experiment was to test whether the selective activation of one (e.g., “left”) out of two contrasting spatial codes (i.e., “left” and “right”) determined the activation of the activation of a corresponding temporal code (e.g., “past”) and vice versa.

### Method

In four different blocks of trials, participants had to (a) go only to left-pointing arrows and to words; (b) go only to right-pointing arrows and to words; (c) go only to past words and to arrows; (d) go only to future words and to arrows. Conditions “a” and “b” qualify the influence of space coding on time coding (space-to-time; i.e., whether the explicit task-induced activation of a spatial code, left or right, facilitates the activation of a corresponding temporal code, past or future, that is not addressed by instructions). Vice versa, conditions “c” and “d” qualify the influence of time coding on space coding (time-to-space; i.e., whether the explicit task-induced activation of a temporal code, past or future, facilitates the activation of a corresponding spatial code, left or right, that is not addressed by instructions). In the space-to-time condition, go temporal targets were intermixed with nonwords no-go nontargets to avoid participants responding to “all” word stimuli without full processing. Nonwords were generated using an online nonword generator (https://www.trainingcognitivo.it/GC/nonparole/) and were equivalent in length and in the number of syllables to target word stimuli (word: 7.07 letters, 3.5 syllables; nonwords: 7.15 letters, 3.5 syllables. All *p*s > .15). In addition, past and future words have the same frequency of use in Italian (*p* > 0.42, http://143.50.35.46/it/cerca) and an equivalent length (past words: 6.75 letters, future words 7.1 letters, *p* > .10). Similarly, in the time-to-space condition, in different trials, arrow targets were alternated with white circle no-go nontargets to promote the full processing of go left- and right-pointing arrows. Each block included 200 trials, with 40 repetitions for each stimulus category.

Go trials in which no response was provided (misses) and trials in which responses were above and below two standard deviations from the mean of each experimental condition were excluded from the analyses. Following these criteria, 3.56% of trials were excluded from analyses.

### Statistical analyses

#### Space-to-time influence

The space-to-time congruency effect was analyzed by entering RTs in congruent trials (past words of condition “a” and future words of condition “b”) and RTs in incongruent trials (past words of condition “b” and future words of condition “a”) in a 2 × 2 repeated-measures ANOVA, with time (past vs. future) and congruency (congruent vs. incongruent) as within-subjects factors.

#### Time-to-space influence

The time-to-space congruency effects were analyzed by entering RTs in congruent trials (left arrow of condition “c” and right arrow of condition “d”) and RTs in incongruent trials (right arrow of condition “d” and left arrow of condition “c”) in 2 × 2 repeated-measures ANOVA, with arrow direction (left vs. right) and congruency (congruent vs. incongruent) as within-subjects factors.

#### Reliability of congruency effects

The reliability of congruency effects was examined using the split-half method. We first divided each experimental condition data into odd and even responses. Then we calculated the RTs advantages produced in the congruent respect to the incongruent condition (dRTs = RTs in the incongruent condition minus RTs in the congruent condition) for the odd-numbered and the even-numbered items. We then evaluated the correlation between the RTs advantages for odd and even-numbered items (coefficient *r*_1, 2_). Besides, we used the corrected Spearman–Brown correlation between the RTs advantages in the odd-numbered and even-numbered halves of trials as the reliability index (coefficient: *r*_tt_). To further assess the reliability of Congruency effects, the split-half test was also applied to 10,000 permutations of RTs (the results of these control analyses are detailed in the Supplementary File).

### Results

#### Space-to-time influence

**Fig. 2:**
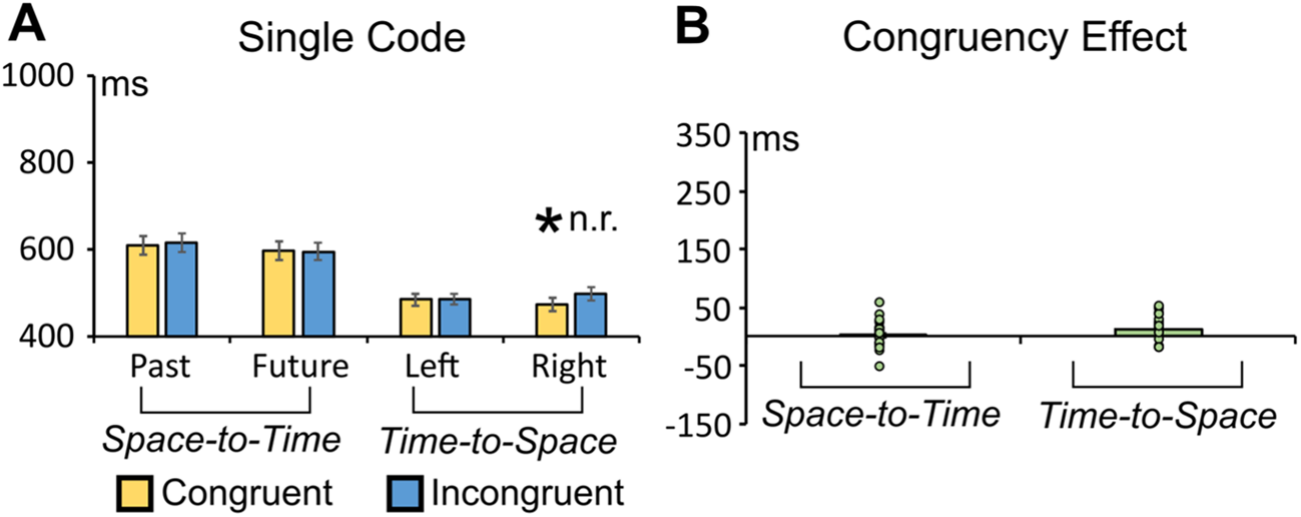
**A** Average RTs in congruent and incongruent conditions in the space-to-time and time-to-space conditions. **B** Congruency effect (incongruent minus congruent RTs difference) in the space-to-time and time-to-space conditions. The asterisk indicates a statistically significant effect (*p* = .01 in this case; the acronym “n.r.” indicates that the effect is unreliable on split-half testing). (Colour figure online)

The ANOVA highlighted no significant main effect of congruency (congruent vs. incongruent), *F*(1, 27) = 0.336, *p* = .566, η_p_^2^ = 0.012, or Time × Congruency interaction, *F*(1, 27) = 0.144, *p* = .70, η_p_^2^ = 0.005; see Fig. 2.

#### Time-to-space influence

The ANOVA revealed a significant Congruency × Arrow Direction interaction, *F*(1, 27) = 5.071, *p* = .032, η_p_^2^ = 0.158 (see Fig. 2). Bonferroni planned comparisons showed significantly faster responses for congruent right-pointing arrow targets (473 ms) versus incongruent right-pointing arrow targets (499 ms; *p* = .013). The same comparison with Left-pointing arrows was not statistically significant (*p* > .562). The reliability test showed inconsistent time-to-space congruency effect (split-half reliability: r_1,2_ = .2453, r_tt_ = .3916, *p* = .20). This result was confirmed when 10,000 random halves were computed through a permutation method (see Supplementary File).

## Experiment 2: Joint code (JC)

Based on the null results of the SC task in Experiment 1, a second go/no-go experiment, hereby defined “joint code” (JC) task, we investigated whether the space–time association is generated when participants are asked to discriminate both the direction of arrow-targets (left vs. right) and the temporal feature of linguistic-targets (past or future), based on instructions that explicitly and fully combine both left and right spatial codes with past/future temporal ones.

### Method

In four different conditions/blocks of trials, participants had to respond (a) only to left-pointing arrows and past words; (b) only to right-pointing arrows and future words; (c) only to left-pointing arrows and future words; (d) only to right-pointing arrows and past words. In conditions “a” and “b” spatial and temporal codes are congruent, while in conditions “c” and “d” they are Incongruent. RTs to temporal-target trials qualified the influence of spatial coding on temporal coding (space-to-time). Vice versa, RTs to arrow-direction trials qualified the influence of temporal coding on spatial coding (time-to-space). Each block consisted of 160 trials, 80 with temporal targets and 80 with arrow targets (40 trials per trial type). Following the same exclusion criteria adopted in Experiment 1, 2.52% of trials were excluded from analyses.

### Statistical analyses

#### Space-to-time influence

The space-to-time congruency effects were analyzed by entering RTs of congruent temporal-target trials (past words in condition “a” and future words in condition “b”) and incongruent temporal-target trials (past words in condition “c” and future words in condition “d”) in a 2 × 2 repeated-measures ANOVA, with Time (past vs. future) × Congruency (congruent vs. incongruent) as within-subjects factors.

#### Time-to-space influence

The time-to-space congruency effects were analyzed by entering RTs of congruent arrow direction-target trials (left arrows in condition “a” and right arrows in condition “b”) and incongruent arrow direction-target trials (left arrows in condition “c” and right arrows in condition “d”) in a 2 × 2 repeated-measures ANOVA, with arrow direction (left vs. right) and congruency (congruent vs. incongruent) as within-subjects factors for the investigation of the time-to-space congruency effect.

#### Reliability of congruency effects

The reliability of congruency effects was examined using the same procedure as in Experiment 1.

### Results

#### Space-to-time influence

**Fig. 3:**
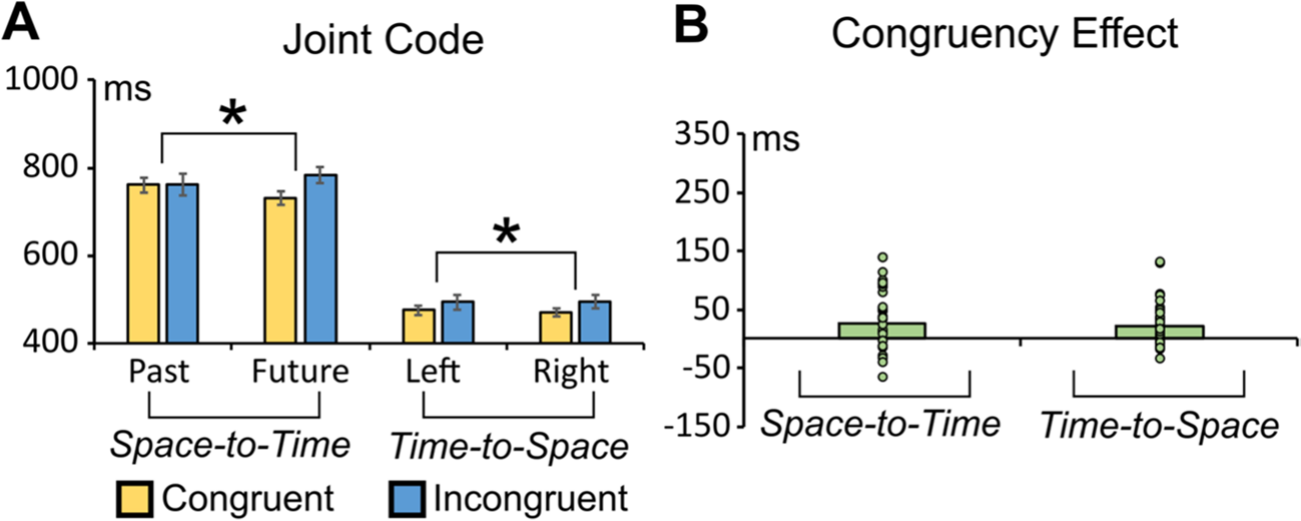
**A** Average RTs in congruent and incongruent conditions, in the space-to-time and time-to-space conditions. **B** Congruency effect (incongruent minus congruent RTs difference) in the space-to-time and time-to-space conditions. Asterisks indicate statistically significant congruency effects (space to time: *p* = .01; time-to-space: *p* = .009). Both effects are reliable on split-half testing (*p* < .001). (Colour figure online)

The ANOVA revealed a significant main effect of congruency, *F*(1, 27) = 7.403, *p* = .011, η_p_^2^ = .215, with faster RTs in the congruent (745 ms) than in the incongruent (772 ms) condition. There was also a significant Congruency × Time interaction, *F*(1, 27) = 17.284, *p* < .001, η_p_^2^ = .390. Bonferroni planned comparisons showed faster responses to congruent future targets (729 ms) versus incongruent future targets (782 ms; *p* < .001). The reliability test showed that the space-to-time congruency effect was consistent (split-half reliability: r_1,2_ = .627, r_tt_ = .771, p = .0003; see Fig. 3]. The result was also confirmed when tested with split-half reliability with 10000 Permutation (see Supplementary File).

#### Time-to-space influence

The ANOVA highlighted a significant main effect of congruency, *F*(1, 27) = 7.899, *p* = .009, η_p_^2^ = .226, with RTs being faster in the congruent (472 ms) than in the incongruent condition (493 ms). Reliability test showed that the time-to-space effect was consistent (split-half reliability: r_1,2_ = .773, r_tt_ = .872, *p* < .0001; see Fig. 3). The permutation test supported the reliability of this result (see Supplementary File).

## Experiment 3: Bimanual STEARC task

The third experiment, hereby defined “bimanual STEARC task,” was designed to test the presence of a conventional STEARC effect in a task that, like the JC one, requires the full combination of left/right spatial codes with past/future temporal one—although, in this case, spatial codes are used to select one out of two possible and spatial defined motor responses.

### Method

Participants performed the task in two different experimental conditions: (a) in the “congruent” condition, they were asked to respond to past words with the left hand/button and future words with the right hand/button; (b) in the “incongruent” condition, vice versa, they were asked to respond to past words with the right hand/button and future words with the left hand/button. Each block consisted of 80 trials, 40 for each past/future condition. The order of blocks was counterbalanced among participants. In this experiment, 4.38% of trials were excluded from analyses due to the exclusion criteria adopted in Experiments 1 and 2.

### Statistical analyses

Individual RTs of the congruent (past-words/left hand-button and future-words/right-hand-button) and incongruent (past-words/right-hand-button and future-words/left-hand-button) conditions were entered in a Time (past vs. future) × Congruency (congruent vs. incongruent) repeated-measures ANOVA.

### Results

**Fig. 4:**
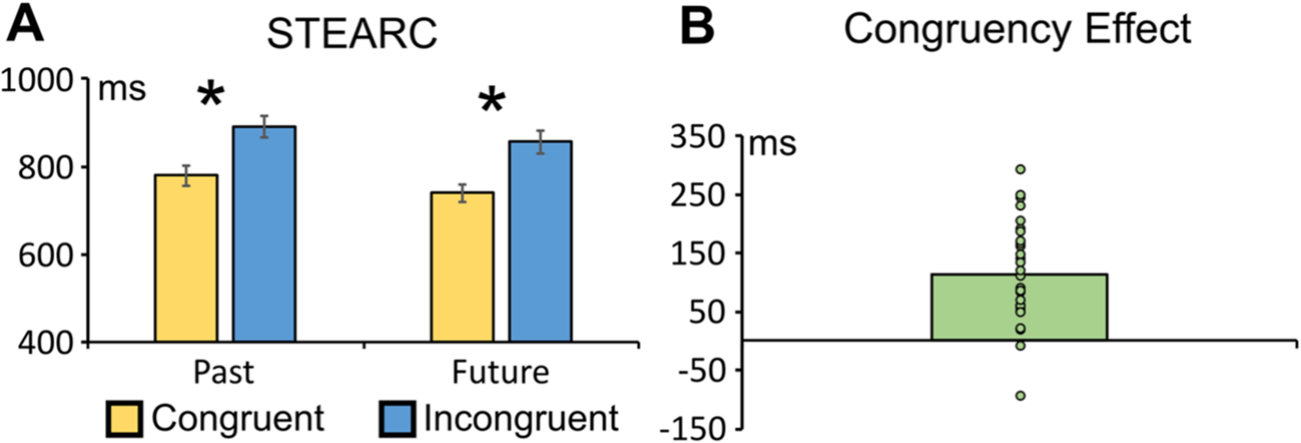
**A** Average RTs to past and future words in the congruent and Incongruent conditions: Asterisks indicate statistically significant congruency effects (*p* < .001). Both effects are reliable on split-half testing (*p* < .001). **B** Average congruency effect. (incongruent minus congruent RTs difference; *p* < .001). (Colour figure online)

The ANOVA highlighted a main effect of congruency, *F*(1, 27) = 50.560, *p* < .001, η_p_^2^ = .651, with faster RTs to congruent (759 ms) than to incongruent targets (873 ms). The reliability analysis confirmed the consistency of the congruency effect (split-half reliability: r_1,2_ = .792, r_tt_ = .884, *p* < .001; see Fig. 4). This result was confirmed when 10,000 random halves were computed through a permutation method (see Supplementary File).

## Comparisons and correlations among space–time congruency effects in Experiments 1, 2 and 3

To compare congruency effects among the three experiments, we ran a one-way ANOVA, with task as the main factor, and a series of correlations. In particular, correlations were run to explore the possible existence of functional relationships among the individual congruency effects observed in the different tasks. To this aim, we tested the correlation between the space-to-time and time-to-space congruency effects observed in the single and joint code tasks with those observed in the bimanual STEARC task.

### Statistical analyses

We computed a series of Pearson-*r* correlations among the individual dRTs (incongruent minus congruent RTs) measured in the three tasks. Preliminary analyses were run to test the presence of multivariate normality in the data set (Raykov & Marcoulides, 2008). The Mahalanobis distance was smaller than the critical value (all *p*s > .001, critical value recommended by Tabachnick et al., 2007), thus showing that no uni- or multivariate outliers were present in our data. In addition, we found that the variable’s distribution was comparable to a multivariate normal (Mardia’s multivariate kurtosis index = 24; *p*-value = 35; Mardia, 1970, 1974).

### Results

**Fig. 5:**
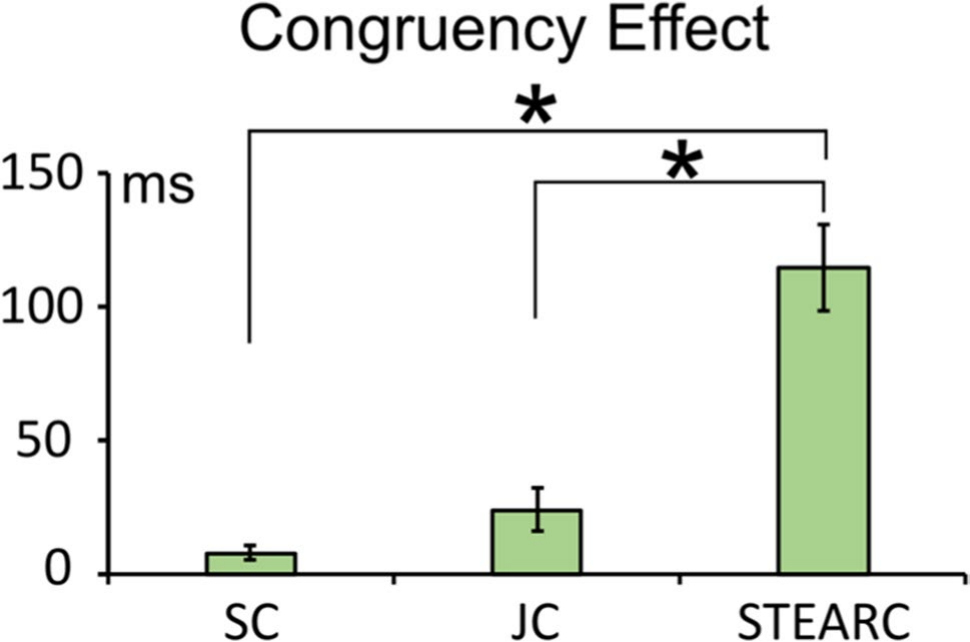
Magnitude of congruency effects (incongruent minus congruent RTs difference) in the single code (SC), joint code (JC), and bimanual STEARC task

The ANOVA pointed out the main effect of task, *F*(2, 54) = 31.060, *p* < .001, η_p_^2^ = .534. Bonferroni planned comparison showed a significant difference between Experiment 1 (SC) and Experiment 3 (STEARC) and between Experiment 2 (JC) and Experiment 3 (STEARC) (all *p*s < .001; see Fig. 5).

Pearson’s correlation (see Table 1) tests highlighted a significant correlation between space-to-time and time-to-space congruency effects observed in the joint code (*p* < .001). Other correlations were not significant (all *p*s > .2; α value corrected by Bonferroni’s formula for multiple comparisons: α_correct_ = .01).

**Table 1:** Results of the correlation analyses

	S-T(sc)	T-S(sc)	S-T(jc)	T-S(jc)	STEARC
S-T(sc)	1				
	*p* = ---				
T-S(sc)	0.0183	1			
	*p* = .926	*p* = ---			
S-T(Jc)	−0.1885	0.0471	1		
	*p* = .337	*p* = .812	*p* = ---		
T-S(Jc)	−0.1846	−0.2193	0.6511 *	1	
	*p* = .347	*p* = .262	*p* = .000	*p* = ---	
STEARC	−0.087	−0.2184	0.1924	0.1686	1
	*p* = .660	*p* = .264	*p* = .327	*p* = .391	*p* = ---

Asterisk indicates a statistically significant effect

## Control Experiment 1: Superordinate category task

Inspired by a variation of the IAT task devised by Pinto and co-workers for the study of SNA (Pinto et al., 2021a, b), in this first control experiment, we investigated to which degree temporal “past vs. future” and spatial “left vs. right” conceptual dichotomies must be explicitly activated by task instructions to generate mental time lines when both temporal and spatial codes are relevant to the performance of the task.

### Method

Thirty-two right-handed Italian native speakers participants (26 F and 6 M, mean age = 23.03 years, *SD* = 3.25) were asked to perform a modified version of the single code experiment. In each condition of the original single code experiment, participants were asked to put in contrast one conceptual dichotomy (e.g., “Go when past words/no go when future words” or “Go when left arrows/no go when right arrows”), while the remaining dichotomy was not activated by task instructions (e.g., “Go to all arrows” or “Go to all words”). In contrast, in this new version of the task, one conceptual code dichotomy was fully activated by task instructions, while the remaining dichotomy was only implicitly activated through its corresponding superordinate semantic code. For example, in the case of spatial codes, “horizontal” is superordinate to “left” and “right.” In the case of temporal codes, “not present-tense” is superordinate to “past” and” future” (see list of “present” words in Supplementary Material). Therefore, in different blocks of trials, participants had to respond (a) only to left-pointing arrows and *not present-tense* words (where past and future words targets were intermixed with present-tense nontargets words); (b) only to right-pointing arrows and *not present-tense* words; (c) only to past words and *horizontal* arrows (where left- and right-pointing arrow targets were intermixed with nontargets vertical arrows that pointed up or down); (d) only to future words and *horizontal* arrows. Conditions “a” and “b” qualify the investigations of space-to-time congruency effects (i.e., whether the explicit task-induced activation of a spatial code, i.e., “left” or “right”) triggers the activation of a corresponding temporal code (i.e., “past” or “future”, i.e., not explicitly activated by instructions). Vice versa, conditions “c” and “d” qualify the investigation of time-to-space congruency effects (i.e., whether the explicit activation of a temporal code, “past” or “future,” elicits the activation of a corresponding spatial code, “left” or “right”) that is not explicitly activated by the task instructions. The procedure of the superordinate category task was similar to that of the single code experiment except that, in this case, vertical up or down pointing arrows for conditions “a” and “b” and present-tense words were also for condition “c” and “d” presented as no-go nontargets. Each block included 200 trials, with 40 repetitions for each category of stimuli. Participants were tested in a quiet and isolated room. They wore in-ear plug headphones to reduce environmental noise. The head position was restrained with a chin rest at a viewing distance of 57.7 cm from the screen. All participants had a normal or corrected-to-normal vision and were naive to the aim of the study.

### Statistical analyses

#### Space-to-time influence

The space-to-time congruency effect was analyzed by entering RTs in congruent temporal trials (past words of condition “a” and future words of condition “b”) and RTs in incongruent temporal trials (past words of condition “b” and future words of condition “a”) in a 2 × 2 repeated-measures ANOVA, with time (past vs. future) and congruency (congruent vs. incongruent) as within-subjects factors.

#### Time-to-space influence

The time-to-space congruency effects were analyzed by entering RTs in congruent spatial trials (left arrow of condition “c” and right arrow of condition “d”) and RTs in Incongruent spatial trials (right arrow of condition “d” and left arrow of condition “c”) in 2 × 2 repeated-measures ANOVA, with arrow direction (left vs. right) and congruency (congruent vs. incongruent) as within-subjects factors.

#### Reliability of congruency effects

The reliability of congruency effects was examined using the same procedure as in Experiment 1.

### Results

#### Space-to-time influence

**Fig. 6:**
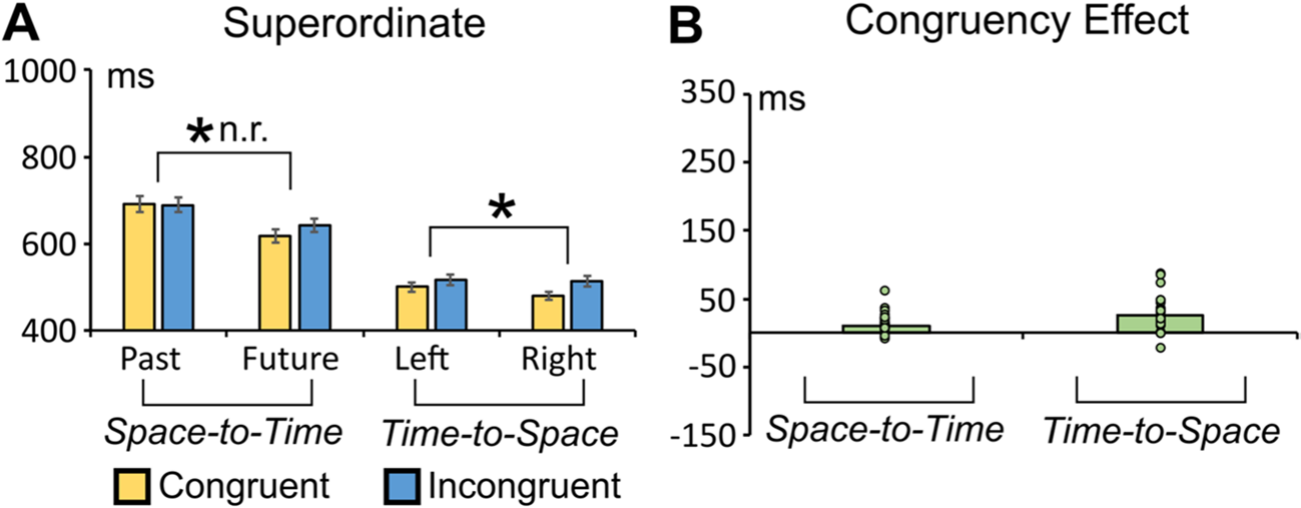
**A** Average RTs in the congruent and incongruent space-to-time and time-to-space conditions of the superordinate category task. **B** Congruency effects (incongruent minus congruent RTs difference) in the space-to-time and time-to-space conditions. The asterisk indicates a statistically significant effect (*p* = or < .001 in this case; the acronym “n.r.” indicates that the effect is not reliable on split-half testing). (Colour figure online)

The ANOVA pointed out a significant main effect of congruency (congruent vs. incongruent), *F*(1, 31) = 12.939, *p* = 0.001, η_p_^2^ = 0.294, with faster RTs to congruent trials (654 ms) than to incongruent ones (664 ms). Nonetheless, the reliability test showed this effect was inconsistent [split-half reliability: r_1,2_ = −.2563, r_tt_ = −.407, p = .156; see Fig. 6]. Also, the permutation method highlighted the non-reliability of this effect (see Supplementary File).

#### Time-to-space influence

The ANOVA highlighted a significant main effect of congruency (congruent vs. incongruent), *F*(1, 31) = 29.175, *p* < .001, η_p_^2^ = 0. 484. Also, in this case, RTs were faster in the congruent (488 ms) than in the incongruent condition (514 ms). The reliability test showed that this effect was consistent (split-half reliability: r_1,2_ = −.544, r_tt_ = −.704, *p* = .001; see Fig. 6). The result was also confirmed when split-half reliability was tested with 10,000 permutations (see Supplementary File).

## Control Experiment 2: Testing the influence of the activation of temporal and spatial codes in the performance of a speeded primary go/no-go and a concomitant nonspeeded secondary classification task

The single code task of Experiment 1 highlighted no space–time interaction. Nonetheless, it could be argued that in Experiment 1, when the instruction was, for example, “Go to left-pointing arrows and to all words,” notwithstanding a distinction between go-words and no-go-nonwords was required by the task, a proper distinction between “past” and “future” words was not made, and these two-word categories were lumped into a more general “word” category to be contrasted with the nonword one. In the same vein, merely responding to all arrow targets and not responding to circles (e.g., “Go only to past words and to arrows”) did not trigger a proper distinction between left- and right-pointing arrows, so that all arrows were lumped in the more general category “arrow” to be contrasted with the category “circle.” Based on these premises, in a second control experiment we examined the space–time association when (a) a primary speeded go task required attending only to “past” or “future” words and a secondary nonspeeded task required classifying the direction of horizontal arrows; (b) a primary speeded go task required attending only to “left” or “right” pointing arrows and a secondary nonspeeded task required classifying words as “past” or “future” ones. For example, when the primary instruction was “Go to left-pointing arrows and to all words,” a second instruction asked participants to “push the green button if the word was a ‘past’ one and the orange button if it was a future one.” In this case, there is no joint space–time code for the primary response though in the secondary task participants are obliged to make an explicit distinction between “past” and “future” words.

### Method

**Fig. 7:**
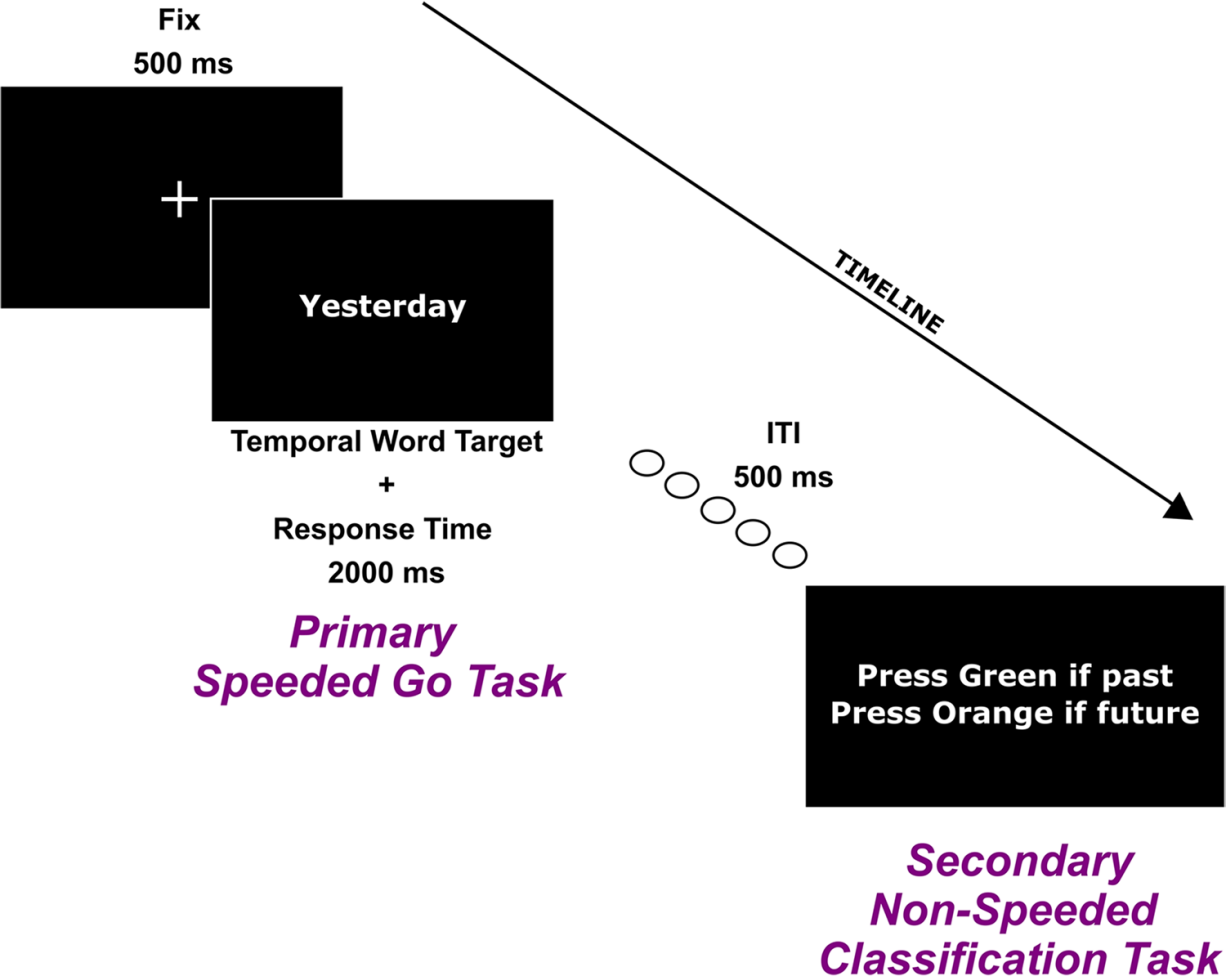
Examples of the two target events included in a go trial of Control Experiment 2. The target (the word “Yesterday” in the example) requires first a speeded go response with word-targets (e.g., instruction “Go only to left-pointing arrows and to all words) and then a second nonspeeded classification of word-targets (e.g., “Press green if past, press orange if future”)

In this second control experiment we tested 28 right-handed Italian native speakers (20 F and 8 M, mean age = 23.1 years, *SD* = 2.55. In different experimental conditions, participants were asked to (a) “go” as fast as possible only to left-pointing arrows and to all words and then indicate, without time pressure, whether the word was a “past” or a “future” one by pressing the “green” or the “orange” button, respectively; (b) “go” as fast as possible only to right-pointing arrows and to all words, and then indicate, without time pressure, whether the word was a “past” or a “future” one by pressing the “green” or the “orange” button, respectively; (c) “go” as fast as possible only to past words and to all arrows and then indicate, without time pressure, whether the arrow was a left- or a right-pointing one by pressing by pressing the “green” or the “orange” button, respectively; (c) “go” as fast as possible only to future words and to all arrows and then indicate, without time pressure, whether the arrow was a left- or a right-pointing one by pressing the “green” or the “orange” button, respectively. Like in the other experiments, the central space bar was used for the primary speeded task. Buttons “B” and “Y” were used for the secondary nonspeeded task: These buttons are vertically arranged one above the other on the computer keyboard and were chosen not to include or suggest the implicit use of left versus right spatial response codes. Half of the participants had the “B” button covered with a green plastic button and the “Y” button with an orange plastic button, while for the other half of participants the position of green and orange buttons was reversed. Conditions “a” and “b” qualify space-to-time congruency effects, while conditions “c” and “d” qualify time-to-space congruency effects. The procedure and stimuli were as in Experiment 1, with the additional inclusion of the secondary nonspeeded task (see Fig. 7). The order of administration of experimental blocks/instructions was counterbalanced among participants. Participants were tested with the same apparatus as the first control experiment.

### Statistical analyses

#### Space-to-time influence

The space-to-time congruency effects were analyzed by entering RTs in congruent temporal trials (past words of condition “a” and future words of condition “b”) and RTs in Incongruent temporal trials (past words of condition “b” and future words of condition “a”) in a 2 × 2 repeated-measures ANOVA, with time (past vs. future) and congruency (congruent vs. incongruent) as within-subjects factors.

#### Time-to-space influence

The time-to-space congruency effects were analyzed by entering RTs in congruent spatial trials (left arrow of condition “c” and right arrow of condition “d”) and RTs in Incongruent spatial trials (right arrow of condition “d” and left arrow of condition “c”) in 2 × 2 repeated-measures ANOVA, with arrow direction (left vs. right) and congruency (congruent vs. incongruent) as within-subjects factors.

#### Reliability of congruency effects

The reliability of congruency effects was examined using the same procedure as in Experiment 1.

### Results

#### Space-to-time influence

**Fig. 8:**
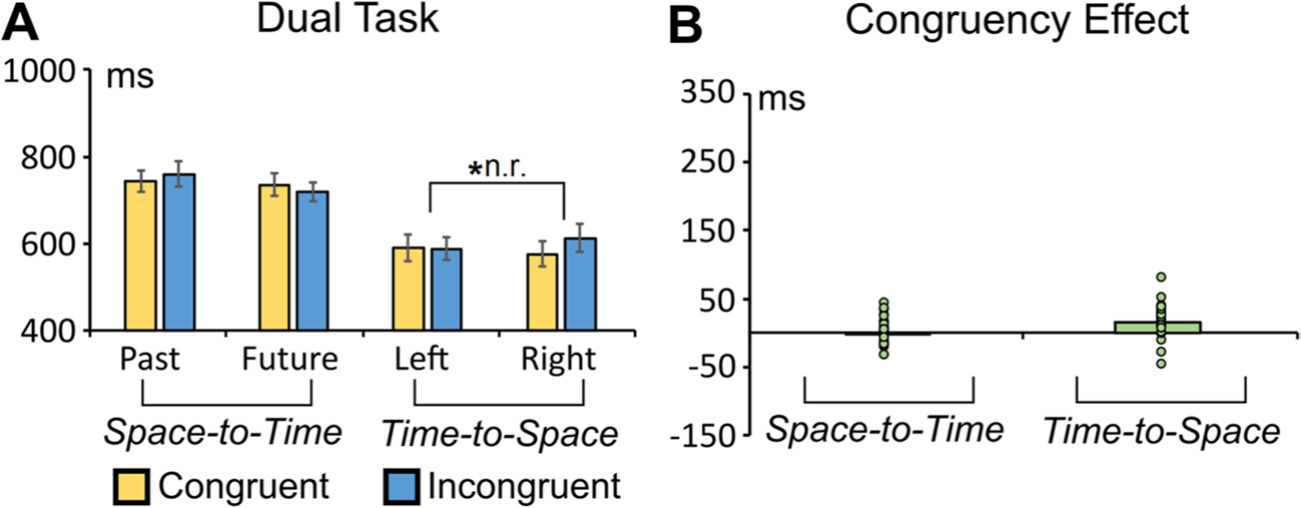
**A** Average RTs in the congruent and incongruent space-to-time and time-to-space conditions of the speeded primary go/nonspeeded secondary classification control task. **B** Congruency effects (incongruent minus congruent RTs difference) in the space-to-time and time-to-space conditions. The asterisk indicates a statistically significant effect (*p* < .01 in this case; the acronym “n.r.” indicates that the effect is not reliable on split-half testing). (Colour figure online)

The ANOVA pointed out no significant main effect of congruency (congruent vs. incongruent), *F*(1, 27) = 0.057, *p* = .813, η_p_^2^ = 0.002 (see Fig. 8).

#### Time-to-space influence

The ANOVA highlighted the main effect of congruency (congruent vs. incongruent), *F*(1, 27) = 10.477, *p* = 0.003, η_p_^2^ = 0. 279. RTs were faster in the congruent (558 ms) than in the incongruent condition (573 ms). Nonetheless, the reliability test showed that this effect was inconsistent (split-half reliability: r_1,2_ = .03, r_tt_ = .05, *p* = .85; see Fig. 8). This result was confirmed by split-half reliability with 10,000 permutations (see Supplementary Material).

## Discussion

The main results of our study are as follows. First, in a single code task (SC; Experiment 1) in which spatial “left” or “right” oriented arrow targets are alternated with “past” or the “future” temporal-word targets, selectively attending to one spatial target/code, “left” or “right”, does not entail the activation of the corresponding “past” or “future” temporal code and vice versa. In Experiment 1, no space–time association (STA) was found except for a statistically unreliable speeding-up in the detection of right-pointing arrows when participants selectively attended to “future” word targets (time-to-space influence). These results were replicated in the second control experiment, which explicitly required holding in mind one spatial or temporal code for selecting a primary speeded go response and explicitly classifying through a secondary nonspeeded response the identity of no-go targets in the other temporal or spatial dimension. This shows that the absence of significant and reliable space–time congruency effects in Experiment 1 was not due to the fact that no discrimination was required between different categories of no-go targets in the temporal or spatial dimension.

Second, in a joint code task (JC; Experiment 2) that required attending to a “left” or “right” spatial code and to a “past” or “future” temporal code at the same time, the STA became significant and reliable. In this case, faster classification of spatial and temporal features of target stimuli was found when instructions combined congruent (i.e., left/past and right/future) rather than incongruent (i.e., left/future and right/past) spatial and temporal codes. In this task, the size of time-to-space facilitatory effects was positively correlated to the size of space-to-time facilitatory effects (for similar results in the SNA, see Pinto et al., 2019a, b, 2021a, b). In line with the results of our previous studies in the SNA (Pinto et al., 2019a, b, 2021a, b), the findings from Experiment 1 and Experiment 2 show that a significant and reliable spatial representation of time is generated only when the task requires the joint and explicit activation of both temporal (i.e., “past”/“future”) and spatial (“left”/“right”) semantic dichotomies. Santiago et al. (2011) have suggested that acquiring new conceptual metaphors requires that two semantic dimensions are “simultaneously included in an active mental model.” Here we expand on this conclusion by showing that also the recovery of a stable and reliable “metaphoric” spatial representation of the flow of time requires the concomitant and explicit activation of both temporal and spatial semantic codes.

Third, the STA showed a dramatic rise in a conventional bimanual STEARC task (Experiment 3) when the association between spatial and temporal codes regulated the selection between a left-side and a right-side motor response. Taken together, the results from Experiments 1 and 3 show that the STA is stronger when the association of spatial and temporal codes is used to select a spatially defined motor response (Experiment 3) rather than when the same association is used to discriminate the spatial and temporal features in alternating arrows and word targets (Experiment 2). The significant STEARC in Experiment 3 replicates the results by Santiago et al. (2007).

Available evidence with event-related potentials (ERP) shows that the STEARC arises at the response-related stage (i.e., during the selection between a left and a right motor response; Vallesi et al., 2011). One possibility to explain the rise of the STA in the STEARC compared with the JC task is that response selection in the STEARC amplifies STA effects already present at the conceptual level and tapped by the JC task. Nonetheless, a series of correlation analyses revealed no significant correlation between the strength of the STA in the JC task and the strength of the STEARC. This finding suggests that the STA highlighted in the JC of the IAT and the STEARC might rely on different mechanisms or different modes of representing time (for a similar dissociation in the number domain, see van Dijck & Doricchi, 2019)

It is interesting to note that even if not triggered by explicit task instructions, as it happens in the STEARC task, the use of directionally contrasting left vs. right (or backward vs. forward) gestures can be spontaneously adopted during the interpersonal communication of temporal information (Núñez & Sweetser, 2006). This observation suggests that the STEARC task and the culturally shaped interpersonal communication of temporal concepts share common cognitive operations. We argue that left/right spatial “affordances” are present in both cases. By “affordance” we mean the properties of an object that define its possible uses (Gibson, 1977). In the STEARC task, the position of the two response buttons offers these affordances. In contrast, the same affordances are offered in interpersonal communication by the symmetrically organized left/right body structure. In the second case, the culturally defined link between the spatial organization of time gestures and reading-scanning habits triggers the use of left/right-hand gestures. In this sense, the unimanual go/no-go task could be interpreted as a task where instructions (i.e., push/don’t push the central button) do not offer left/right spatial affordances and, as a consequence, do not promote the activation of a reliable and spatially organized MTL. We conclude that both in the case of the STEARC and interpersonal communication, it is the possibility of using spatially contrasting left/right spatial-motor codes that induce the spatial left/right mental representation of time flow—that is to say that the spatial representation of time is contingent upon the environmental/task set, upon the heuristic value of representing time through space and not always necessarily inherent to time representation. These conclusions are supported by developmental studies showing that the left-to-right representation of time appears gradually in 5-year-old children (Tillman et al., 2022) and stabilizes at 8–10 years of age (Droit-Volet & Coull, 2015).

The results of our study are congruent with and expand on the results of previous investigations. Weger and Pratt (2008) studied the spatial effects produced by retrospective (e.g., “yesterday”) and prospective (e.g., “tomorrow”) central word cues on the speed of detection of ensuing visual targets presented on the left or the right side of space. When the side of target presentation had to be classified through spatially corresponding left versus right button presses, responses to left-side targets were faster with retrospective cues and right-side targets with prospective ones. However, and entirely in line with the conclusions of our study, the same STA was not observed when visual targets had to be merely detected through a unimanual central response. Anelli et al. (2018) asked healthy participants and right brain-damaged patients with and without spatial neglect for the left side of space to determine whether personal events were in the past or the future. When the task required selecting a left versus right motor response, a significant STEARC effect was found. In contrast, with vocal non-spatial responses, no STEARC was found in healthy participants and brain-damaged patients. In addition, a striking dissociation between space and time representation was found in patients with neglect because they processed more slowly and less accurately future than past events (i.e., events on the right attended rather than on the left unattended side of mental space).

Interestingly, horizontal MTLs show an important degree of plasticity. For example, Casasanto and Bottini (2014) showed that after brief exposure to mirror-reversed or 90° rotated orthography, left-to-right readers change the direction of their mental timeline from left-to-right to right-to-left. At variance with the horizontal MTLs, which depend on cultural reading and scanning habits, front–back sagittal MTLs are grounded in the functional constraints of forward locomotion and represent a physiologically based and universal phenomenon (Hartmann & Mast, 2012; Rinaldi et al., 2016) that, in some cases, can still be modulated by culturally-based habits (Callizo-Romero et al., 2022). Given the likely different functional origins of horizontal and sagittal MTLs, future studies should assess whether the findings from the present study also extend to the sagittal front-back spatial representation of time.

To conclude, the results of our study point out the important role that the explicit interplay between spatial and temporal codes has in the genesis of stable and reliable MTLs. The progressive increase in the size and reliability of space–time congruency effects that we have observed when passing from the SC and JC to the STEARC task suggests that it is helpful to consider the mental–spatial representation of time as being not an all-or-none event but instead, as an event whose strength can vary as a function of environmental and task conditions. As an example, in the superordinate category task experiment, participants had to respond to targets according to instructions that fully activated one conceptual contrast in the spatial or temporal dimension (e.g., “Go only to ‘present’ words”) while the contrast in the remaining dimension was only implicitly activated through a superordinate semantic code (e.g., “Go when the arrow is horizontal”; horizontal is superordinate to “left” and “right” pointing arrows). In this case, we found significant congruency effects in the Space-to-Time and Time-to-Space directions (although only the latter resulted in being reliable).

Current research mainly focuses on qualifying the types and the set of sensorimotor and cultural experiences that shape the spatial representation of abstract concepts like time and number. For example, Pitt and Casasanto (2020) have suggested that the horizontal mental spatial representations of time and numbers might rely on non-entirely overlapping sets of “sensorimotor experiences.” This was demonstrated by showing that counting backwards from 10 to 1, starting from the right thumb and ending with the left thumb, leaves unchanged the mental position of ascending numbers from left-to-right in the MNL tough, at the same time, reverses the direction of the mental flow of time, because in this case large numbers are associated with past events in the right side of space. Studies like that of Pitt and Casasanto (2020) highlight the importance of investigating the set and the functional hierarchy of sensory-motor and cultural experiences that determine the spatialization of abstract concepts like numbers and space. Nonetheless, we think that another relevant and perhaps even more fundamental challenge in understanding the functional bases of MTLs, is to define the behavioural conditions that trigger their activation and use in everyday life: the present study aims to provide new clues for clarifying this issue.

In the domain of space-number association, recent investigations have suggested that interindividual variations in scanning habits, finger counting style and spatial imagery can significantly affect the ability to generate and use mental spatial representations of numerical magnitudes (Fischer & Knops, 2014; Pellegrino et al., 2019): A challenge for future studies will be to assess whether similar interindividual variations also affect the ability to generate and use spatially organized MTLs.

### Supplementary Information

Below is the link to the electronic supplementary material.

Supplementary file1 (DOCX 124 KB)

## Data Availability

The datasets generated during and/or analyzed during the current study are available from the corresponding author upon reasonable request.

## Footnotes

1 Please note that our conclusions deal with the processing of single numbers, as the processing of ordered series of numerical and nonnumerical items in working memory can be endowed with a spatial component independently from the use of spatial response codes (see van Dijck et al., 2014).
