# Supplementary material for: How time gets spatial: factors determining the stability and instability of the mental time line
Source: Atten Percept Psychophys. 2023 Jul 19;85(7):2321–36. doi: 10.3758/s13414-023-02746-w (PMC10584722; doi:10.3758/s13414-023-02746-w)
Supplement: Supplementary file 1 — Supplementary file1 (DOCX 124 KB) [file 13414_2023_2746_MOESM1_ESM.docx]

**Supplementary Material**

**How time gets spatial: factors determining the stability and instability of the Mental Time Line**

*Gabriele Scozia ^1,3*^, Mario Pinto ^1^, Michele Pellegrino ^1^, Silvana Lozito ^1,3^, Lorenzo Pia ^5^, Stefano Lasaponara ^1,4^, and Fabrizio Doricchi^1,2 *^*

*^1^ Dipartimento di Psicologia, Università degli Studi di Roma ‘La Sapienza’, Roma, Italy*

*^2^ Fondazione Santa Lucia IRCCS, Roma, Italy*

*^3^ PhD program in Behavioral Neuroscience, Università degli Studi di Roma ‘La Sapienza’, Roma, Italy*

*^4^ Libera Università Maria Santissima Assunta – LUMSA, Roma, Italy*

***^5^*** *Department of Psychology, University of Turin, Turin, Italy*

*^*^Corresponding authors:* [*fabrizio.doricchi@uniroma1.it*](mailto:fabrizio.doricchi@uniroma1.it)*,* [*gabriele.scozia@uniroma1.it*](mailto:gabriele.scozia@uniroma1.it)

**Full set of words and non-words**

| **Past** | **Future** | **Present** | **No-Words** |
| --- | --- | --- | --- |
| Passato | Futuro | Presente | nebuse |
| Prima | Dopo | Ora | fecedo |
| Recentemente | Prossimamente | Attualmente | bofate |
| Ieri | Domani | Oggi | zipalore |
| Disse | Dirà | Dice | vizapi |
| Ebbe | Avrà | Ha | peploni |
| Parlai | Parlerò | Parlo | gaplive |
| Potemmo | Potremo | Possiamo | vegreva |
| Cercaste | Cercherete | Cercate | liprure |
| Provaste | Proverete | Provate | maslozi |
| Andasti | Andrai | Vai | fecuogri |
| Apparii | Apparirò | Appaio | ebridono |
| Vide | Vedrà | Vede | ovruconi |
| Pensai | Penserò | Penso | legutoia |
| Guardai | Guarderò | Guardo | gligorifo |
| Chiese | Chiederà | Chiedo | narge |
| Decideste | Deciderete | Decide | parobronteca |
| Facesti | Farai | Fai | unfa |
| Credesti | Crederai | Credi | lideuracro |
| Guidammo | Guiderai | Guidiamo | cluta |

**Table 1.**

**Testing Split-Half Reliability with Permutation Test**

In the analyses reported in the manuscript, the reliability of Congruency effects was examined using the split-half method. We first divided each experimental condition data into odd and even responses. Then we calculated the RTs advantages produced in the Congruent respect to the Incongruent Condition (dRTs = RTs in the Incongruent Condition minus RTs in the Congruent Condition) for the odd-numbered and the even-numbered items. We then evaluated the correlation between the RTs advantages for odd and even-numbered items (coefficient r_1,2_). Besides, we used the corrected Spearman-Brown correlation between the RTs advantages in the odd-numbered and even-numbered halves of trials as an index of reliability (coefficient: r_tt_).
To further improve the stability of our results we examined the reliability of Congruency effects of the different experiments report in the study using a Permutation method by computing 10000 random halves. We then compared the orgianal reliability coefficient obtained in the odd-even split half method within the distribution of 10000 different reliability coefficients, in order to calculate the p-value after the permutation. We plotted the results in a histogram and put a vertical line at our original observed correlation. The p-value was computed dividing the number of coefficient larger than the original reliability coefficient by the number of permutations i.e., 10000.

**Results
*Experiment 1: Single Code***

**
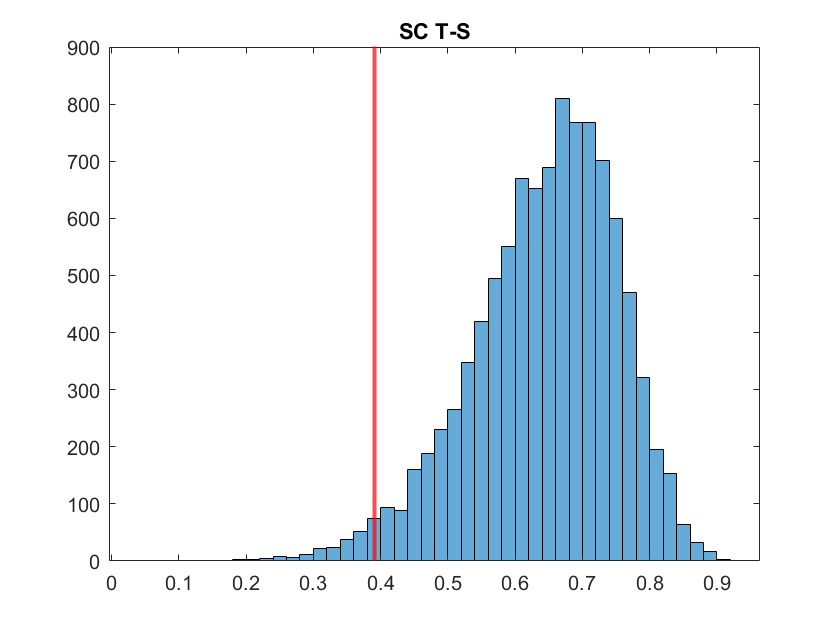
**

Time-to-Space: r_1,2_ = .245, r_tt_ = .391, p = .97.

***
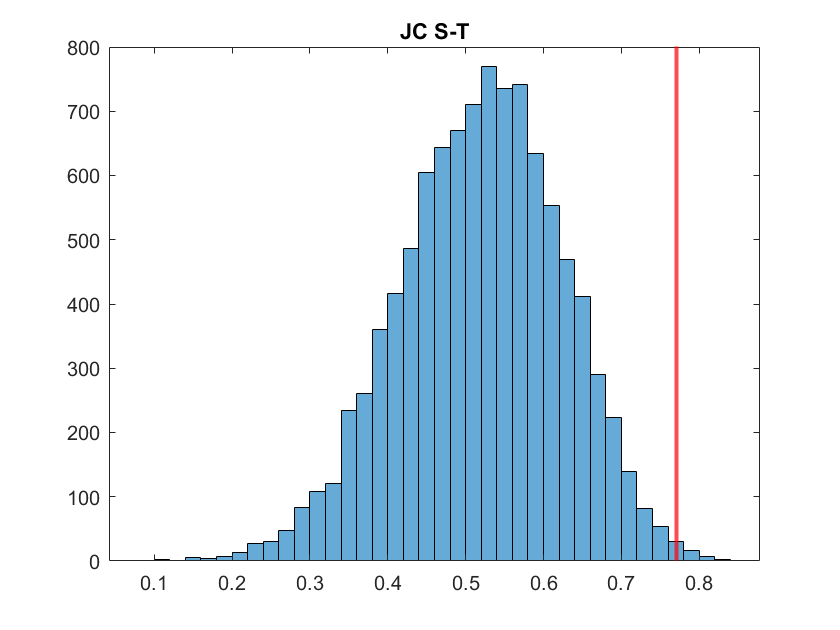
Experiment 2: Joint Code***

Space-to-Time: r_1,2_ = .627, r_tt_ = .771, p = .0043.


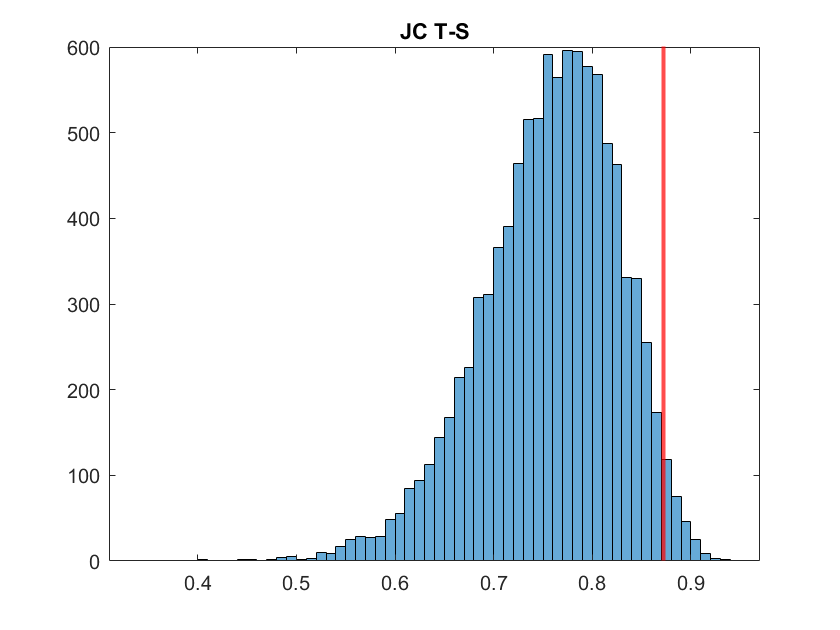


Time-to-Space: r_1,2_ = .773, r_tt_ = .872, p = .0246.

***Experiment 3: Bi-manual STEARC task***

***
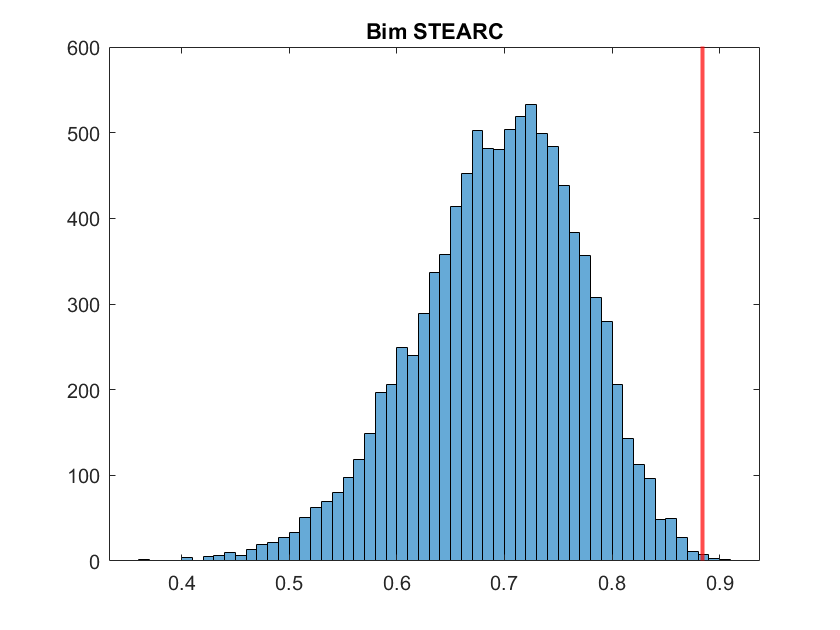
***

STEARC: r_1,2_ = .792, r_tt_ = .884, p = .0007

***Control Experiment 1: Superordinate Category Task***

***
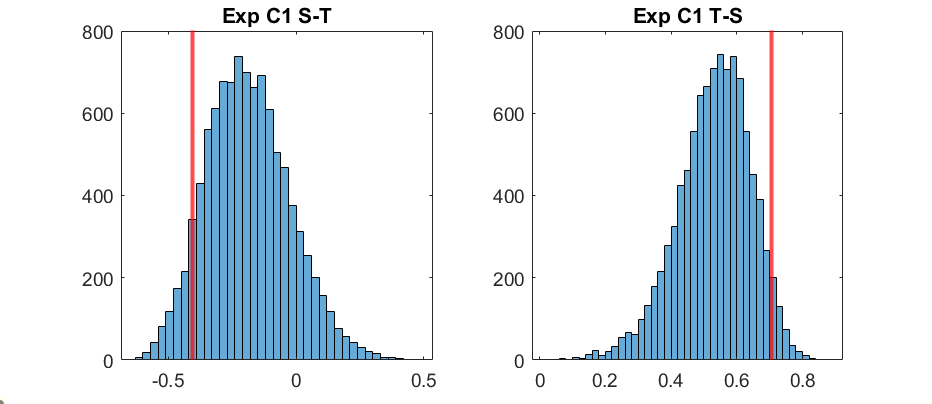
***

Space-to-Time: r_1,2_ = -.256, r_tt_ = -.407, p = .9217;
Time-to-Space: r_1,2_ = .544, r_tt_ = .704, p = .0422.

***Control Experiment 2: Testing the influence of irrelevant codes for Go responses in a dual task.***


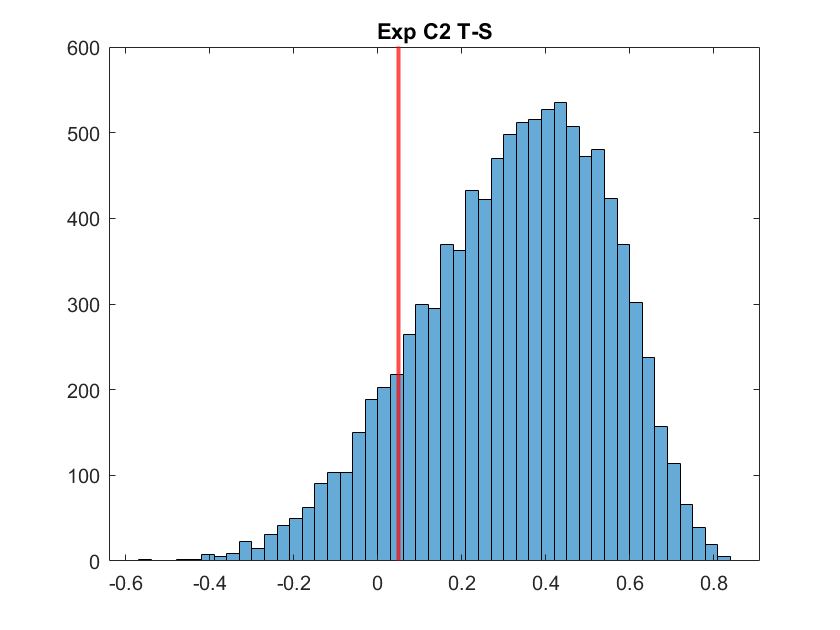


Time-to-Space: r_1,2_ = .03, r_tt_ = .05, p = .877.

**Conclusion**Taken together the analyses performed with 10000 Permutations confirm and enhance the results of the reliability coefficients obtained with odd-even split-half method.
